# Dietary *Moringa oleifera* Alters Periparturient Plasma and Milk Biochemical Indicators and Promotes Productive Performance in Goats

**DOI:** 10.3389/fvets.2021.787719

**Published:** 2022-03-03

**Authors:** Ali Afzal, Tarique Hussain, Amjad Hameed, Muhammad Shahzad, Muhammad Usman Mazhar, Guan Yang

**Affiliations:** ^1^Animal Sciences Division, Nuclear Institute for Agriculture and Biology College, Pakistan Institute of Engineering and Applied Sciences (NIAB-C, PIEAS), Faisalabad, Pakistan; ^2^Nuclear Institute for Agriculture and Biology College, Pakistan Institute of Engineering and Applied Sciences (NIAB-C, PIEAS), Faisalabad, Pakistan; ^3^Department of Infectious Diseases and Public Health, City University of Hong Kong, Kowloon, China

**Keywords:** *Moringa oleifera*, plasma biochemistry, antioxidant status, milk composition, productive performance, goat

## Abstract

The purpose of the current study was to explore the supplementing effects of *Moringa oleifera* leaf powder (MOLP) on plasma and milk biochemical indices and productive/reproductive performance of goats. A total of 30 healthy pregnant goats were randomly distributed (*n* = 10) into three experimental groups: control (M_0_) group (basal diet without MOLP), M_2%_ group (basal diet + 2% MOLP), and M_3.5%_ group (basal diet + 3.5% MOLP). The experiment started 2 months before parturition and continued till the first month of lactation. The plasma flavonoids were significantly increased in the M_3.5%_ group during the entire experiment, whereas the total phenolic contents were enhanced only during the lactation period depending on the supplementation percentage. The amount of vitamin C increased significantly in M_2%_ and M_3.5%_ groups as compared to the M_0_ group. Supplementation of MOLP improved the plasma total antioxidant capacity by declining malondialdehyde concentration and total oxidant status values. The activities of superoxide dismutase and peroxidase enzymes were modified in M_2%_ and M_3.5%_ supplemented groups throughout the experiment, while the catalase activity was significantly influenced only during the lactation stage. The protein and lycopene contents in plasma were significantly improved in the M_3.5%_ group, whereas the total sugars and carotenoid level was increased in both M_2%_ and M_3.5%_ groups. Dietary supplementation with 3.5% MOLP more effectively enhanced protease and amylase activities as compared to 2% supplementation. MOLP also improved the biochemical indices and antioxidant status of colostrum and milk. The milk yield, weight gain of the kids, and reproductive performance were high in M_2%_ and M_3.5%_ groups in comparison to the M_0_ group. These findings disclose that supplementing the diet with 3.5% MOLP improves antioxidant status, milk yield, and reproductive performance in goats.

## Introduction

Reproductive performance is a key factor in goat production and is directly related to maternal nutrition. The pariparturient period (late gestation and early lactation) is characterized by depressed feed intake, endocrine, and metabolic changes that interrupt energy balance and anti-oxidant status of the body (1, 2). Nutritional requirements are high during this period due to accelerated digestion rate, tissue mobilization for mammary development, and fetus growth (3). However, maternal malnutrition is common in small ruminants in most parts of the world due to scarcity and high cost of feed stuffs. The farmers, in these regions, mostly depend on conventional grazing and crop residues to meet the requirements of their animals. The natural grazing pastures and crop residues have fluctuating nutritional status, and their feeding alone is not sufficient to satisfy the energy needs of pregnant and lactating animals (4). The energy deficit feed makes the pregnant animals more prone to oxidative stress with enormous production of reactive oxygen species (ROS) that results in a disturbance of the balance between oxidant and antioxidant defense systems of the body (5). All biomolecules including lipids, carbohydrates, and proteins are adversely affected by oxidative stress, which ultimately leads to a decline in reproductive and productive performance (6). Furthermore, the newborn kids may suffer from a variety of diseases that will negatively influence their survival and growth rate (7).

Colostrum is the first secretion produced soon after parturition and is a source of immunity for newborns. Immunoglobulins, minerals, and many other biologically active substances are transferred passively through the colostrum to the kids of sheep, goat, cattle, and horse as they do not get into the embryo's bloodstream (8). The composition and quality of colostrum and milk predominantly depend on the nutrition of the mother (9, 10). The feed should fulfill the nutritional requirements of pregnant animals to get good quality of colostrum and milk from them after parturition. The inadequate supply of nutrients will adversely affect the synthesis and composition of colostrum and milk.

The diet manipulation with phytobiotics (plant-derived feed additives) has been proposed to be an effective approach in managing nutrition-induced oxidative stress during pregnancy and lactation in both small and large ruminants (11–14). Some herbal plants have medicinal values and are nutritionally important to enhance the health status and reproductive performance of goats. Their supplementation with a basal diet can minimize nutrition-related problems in goat production (15).

*Moringa oleifera* (MO) is an evergreen tree fodder, also known as a “miracle tree,” and is one of the most useful, multi-purpose, fast-growing, and drought-resistant trees. It is well-known for its medicinal importance and nutritional characteristics. Moringa leaves contain a sufficient quantity of vitamins, minerals, and proteins according to the nutritional demands of pregnant and lactating animals (16, 17). Moreover, MO leaves are also a rich source of different bioactive compounds, especially abundant in antioxidants including flavonoids (kaempferol, myricetin, and quercetin), phenolic acids (gallic, ellagic, and chlorogenic acid), Vit C, Vit E, β-carotene, Se, and Zn (18). These substances have been detected separately in various plants, but MO is distinct in having them all in substantial amounts (19).

The MO leaves are readily adapted and easily digested by animals. The favorable impacts of MO have been observed on the anti-oxidant status and reproductive performance in mice and sows (20, 21). However, there is little information about dietary supplementation effects of *Moringa oleifera* leaf powder (MOLP) during the nutritionally critical stages (late pregnancy and early lactation) in goats. Therefore, the study was designed to evaluate the effects of MOLP as a nutritional supplement on productive and reproductive performance, plasma, and milk biochemical indices in Beetal goats. The results of this study will enlighten the knowledge about the development of different bioactive compounds from MO leaf in the field of goat reproduction.

## Materials and Methods

### Ethical Statement

The procedures used for study and ethical clearance were approved and granted by Animal Use and Care Research Committee at Nuclear Institute for Agriculture and Biology (NIAB), Faisalabad, Pakistan.

### Experimental Design and Animal Husbandry

The experiment was conducted at the goat research farm of Nuclear Institute for Agriculture and Biology (NIAB), situated ~7 km from the nucleus of the city Faisalabad, Pakistan (longitude 73.0791° E and latitude 31.4287° N) at an altitude of 184 m from sea level. The average rainfall and temperature were 15.50 mm and 41°C during the months (April to July 2020) of the experiment.

A total of 30 pregnant Beetal goats of 2–3 years age and weighing about 40 kg ± 2.3 were selected exactly 2 months before their predicted delivery. The ultrasonographic examination of goats was performed by the Department of Theriogenology, University of Agriculture, Faisalabad for confirmation of pregnancy. All the goats were randomly divided (*n* = 10) into the control (M_0_) group (250 g basal diet without MOLP/animal/day), M_2%_ group (250 g basal diet with 5 g MOLP/animal/day), and M_3.5%_ group (250 g basal diet with 8.75 g MOLP/animal/day). The basal diet was composed of wheat, corn, rice bran, sugarcane molasses, minerals, and soybean meal formulated according to the nutritional requirements of pregnant goats (Table 1) (23). The experiment was initiated 2 months before the estimated due date of kidding and continued till the first month of lactation. All the animals were acclimatized 1 week for the basal diet. Before the start of the experiment, deworming was performed with Albendazole (Zoben, Prix, Lahore, Pakistan) at a dose rate of 2.5 mg/5 kg/BW. The goats were kept in well-ventilated semi-open sheds and offered two times free pasture grazing (*Chloris gayana, Leptochloa fusca, Carduus nutans, Chenopodium album*, and *Cirsium arvense*) during morning and evening schedule with free access to clean drinking water.

**Table 1:** Formulation of basal diet (% DM).

**Ingredients**	**Amount (%)**
Wheat grains	30
Corn	20
Wheat bran	15
Rice bran	15
Wheat straw	10
Soyabean meal	5
Molasses	3
Dicalcium phosphate	1.8
Vitamin and mineral premix^**a**^	0.2
Total	100

a *Vitamin and mineral premix per kg containing: Vit E 300 IU, Vit A 50,000 IU, Vit D3 80,000 IU, Ca 18.5%, Mg 8.2%, P 3.5%, Na 3%, Zn 3,200 mg, Mn 3,333 mg, Cu 800 mg, Se 24 mg, Iodate 68 mg, Co 16 mg (22)*.

### Plant Material

The fresh green leaves of MO (PKM1) were collected from a breeder's farm (Lahore, Pakistan) during the month of November 2019, and their authenticity was assured by an experienced botanist at NIAB. A representative sample of leaves was sent to the institute's herbarium for reference in the future. The leaves were cleaned properly by washing and dried under shade at room temperature for 4 days. The dried leaves were processed to make powder and then stored in airtight containers for use in the experiment.

### Compositional Analysis of Diet

The MOLP and basal diet were subjected to proximate chemical compositional analysis by using standardized methods of analytical chemists (24) as also used in our previous study (22). The MOLP was also analyzed for its different biochemical elements spectrophotometrically (UV-VIS U2800, Hitachi, Japan). The compositional analysis of MOLP and basal diet are presented in Tables 2, 3.

**Table 2:** Chemical composition of basal diet and MOLP (DM basis).

**Constituents**	**Basal diet**	**MOLP**
	**Mean (%) ±SE**	**Mean (%) ±SE**
Dry matter	94.8 ± 0.43	92.5 ± 0.52
Crude protein	13.5 ± 0.16	18.2 ± 0.06
Crude fat	4.4 ± 0.23	5.5 ± 0.05
Ash	16.7 ± 0.37	11.3 ± 0.03
Nitrogen free extract	47.3 ± 0.33	38.4 ± 0.29
Neutral detergent fiber	53.2 ± 0.26	32.4 ± 0.12
Acid detergent fiber	18.06 ± 0.13	19.1 ± 0.08

*MOLP, Moringa oleifera dried leaf powder (22)*.

**Table 3:** Nutritional constituent analysis of MOLP.

**Biochemical constituents**	**Mean ±SE**
Total phenolic contents (μM/g)	36,000 ± 3.21
Total Flavonoids (μg/g)	258.58 ± 2.28
Vitamin C (μg/g)	546.16 ± 3.06
Lycopene (mg/g)	9.95 ± 0.17
Total carotenoids (mg/g)	13.87 ± 0.33
Total sugars (mg/g)	27.51 ± 1.52
Methionine (% of DM)	0.42 ± 0.012
Cysteine (% of DM)	0.52 ± 0.014
Sodium (mg/g)	2.13 ± 0.075
Calcium (mg/g)	180 ± 1.154
Potassium (mg/g)	8.99 ± 0.571
Selenium (mg/g)	0.31 ± 0.057
Iron (mg/g)	0.16 ± 0.034

*MOLP, Moringa oleifera leaf powder (22)*.

### Collection of Blood and Milk Samples

Blood samples (5 ml) were collected in sterile EDTA tubes (Vacutainer, Xinle) from the jugular vein with 20-day intervals after the start of the experimental diet from day 90 of gestation. Plasma was separated from blood samples by centrifugation at 3,000 rpm/4°C and stored at −20°C till further analysis. Colostrum samples were obtained within 2 h after parturition. Milk samples were collected with 1-week interval for 4 consecutive weeks and stored at −20°C for further biochemical analyses. Blood and milk samples were collected at 7–8 a.m. during the whole experiment. The defatting of colostrum and milk samples was done by centrifugation at 2,500 × *g* for 15 min for enzymatic and non-enzymatic antioxidant estimation.

### Analysis of Blood Plasma

#### Non-enzymatic Antioxidants

##### Total Flavonoids

Total flavonoids (TF) in plasma samples were estimated by AlCl_3_ colorimetric assay and rutin was used as standard (25). The samples were mixed with 100 μl of 10% AlCl_3_, 100 μl of 1 M potassium acetate, and 275 μl of deionized water. The contents were incubated for 40 min at room temperature and then absorbance was measured at 415 nm by using a double beam spectrophotometer (UV-VIS U2800, Hitachi, Japan). The TFs were computed with the help of a standard curve and expressed as μg RE (retinol equivalents) per milliliter of sample.

##### Total Phenolic Content

Total phenolic contents (TPC) were assessed using a modified Folin-Ciocalteu procedure (26). The 100-μl blood plasma sample was vortexed with 100 μl of Folin-Ciocalteu reagent for 15 s and then incubated for 1 h at room temperature after adding 700 mM Na_2_CO_3_ (800 μl). The absorbance of reaction was read at 765 nm and TPCs were calculated from a linear regression equation.

##### Vitamin C

Vitamin C was determined by using a standardized protocol of Moeslinger et al. (27). Briefly, vitamin C causes reduction of a colored compound 2,6 Dichlorophenolindophenol (DCPIP) into DCPIPH_2_ (colorless compound). This reaction was monitored by fall-off absorbance at 520 nm. The concentration of vitamin C in plasma samples was measured by using a standard calibration curve.

##### Malondialdehyde

Malondialdehyde (MDA) concentration in blood plasma samples was assessed colorimetrically by using MDA as standard (28). Plasma sample (25 μl) was homogenized in 0.1% trichloroacetic acid and centrifuged exactly for 5 min at 14,000 × *g*. Then, trichloroacetic acid (20%) containing thiobarbituric acid (0.05%) was added in 1 ml aliquot of the supernatants and heated for 30 min by placing in boiling water bath. The reaction mixture was cooled after removing from the water bath and centrifuged for 10 min at 14,000 × *g*. The absorbance of clear supernatants was read at 535 nm and the value of non-specific absorbance (600 nm) was subtracted from it. The MDA contents were measured by a coefficient of extinction 155 per mM per cm.

##### Total Antioxidant Capacity

Total antioxidant capacity (TAC) of plasma samples was measured by an assay based on the reduction of a blue radical cation (ABTS^∙+^) to its original ABTS colorless form by antioxidants (29). The assay solution to measure TAC is composed of reagent R_1_ (CH3COONa buffer and glacial acetic acid), R_2_ (H_2_O_2_, Na3PO4 buffer, glacial acetic acid, and ABTS), and sample. The contents of the reaction solution were incubated for about 6 min at room temperature and then absorbance was read at 660 nm. The TAC value was computed from a standard ascorbic acid calibration curve and represented as ascorbic acid (μM) equivalent per milliliter of sample.

##### Total Oxidant Status

The method for the determination of total oxidant status (TOS) values of plasma samples is based on the oxidation of Fe^2+^ into Fe^3+^ by oxidants present in the sample (29). A specific color appeared when Fe^3+^ ions react with xylenol and the magnitude of color is directly related to the quantity of oxidant molecules that were measured spectrophotometrically. The reaction solution for the determination of TOS value is composed of R_1_, xylenol solution (0.38 g in 500 μl of 25 mM H_2_SO_4_), R_2_ [ferrous ammonium sulfate (II) 0.0196 g, o-dianisidine 0.0317 g, glycerol 500 μl, and NaCl 0.4 g], and sample. The absorbance value was read after 5 min of adding R_2_. Hydrogen peroxide (H_2_O_2_) was used to calculate the final value of TOS that was expressed in μM H_2_O_2_ equivalent per milliliter.

#### Enzymatic Antioxidants

##### Superoxide Dismutase Activity

The plasma samples were assayed for superoxide dismutase (SOD) activity by an inhibition assay that works on the base of SOD ability to inhibit the photochemical reduction of nitroblue tetrazolium (NBT) into formazan (30). The reaction solution for the study of inhibition assay is composed of 50 mM potassium phosphate buffer (pH 7.8), 13 mM L-methionine, 57 μM NBT, Triton X-100 (0.025%), riboflavin (0.004%), and 50 μl of blood plasma sample in a total volume of 3 ml. The photoreaction was performed in a box lined with aluminum (Al) foil and having a 15-W lamp as a light source. The absorbance of the reduction reaction of NBT to formazan was taken at 560 nm and a unit of SOD activity was defined as the amount of enzyme required to cause 50% inhibition of NBT.

##### Peroxidase Activity

Plasma peroxidase (POD) activity was estimated by using the method of Agostini et al. (31) with some necessary modifications and using guaiacol as substrate. The assay solution to measure POD activity contained guaiacol (200 mM), H_2_O_2_ (400 mM), 545 μl of distilled water, 200 mM potassium phosphate buffer (pH 7.0), and 15 μl of blood plasma sample. The reaction was initiated immediately after the addition of the plasma sample, and the absorbance of the reaction solution was measured after every 20 s for 1 min at 470 nm. One unit of POD activity was narrated as the quantity of enzyme that catalyzed the oxidation of guaiacol.

##### Catalase Activity

Catalase (CAT) activity of blood plasma samples was measured by mixing the samples with 50 mM potassium phosphate buffer (pH 7.0) and dithiothreitol (1 mM) as described by Beers and Sizer (32). The reaction mixture to study CAT activity contained 59 mM H_2_O_2_, 50 mM phosphate buffer (pH 7), and a 100-μl plasma sample. The decreasing pattern of absorbance was measured after every 20 s for 1 min at 240 nm and a unit of CAT activity was described as a change in absorbance in 0.01 min.

#### Biochemical Parameters

##### Total Soluble Protein

The quantitative protein estimation of plasma samples was performed by dye-binding method as described by Bradford (33). The plasma sample (5 μl) was mixed and homogenized with 0.1 N NaCl. The reaction solution was incubated for 5 min at room temperature after adding 1 ml Bradford dye to form a protein–dye complex. Thereafter, absorbance was measured at 420 nm.

##### Total Sugar

The total sugar level of plasma samples was enacted by Folin's (34) protocol with few desired modifications. Briefly, the samples were mixed with sulfuric acid (H_2_SO_4_) and neutralized by using sodium carbonate (Na_2_CO_3_). The contents were then filtered and absorbance was read spectrophotometrically at 415 nm for estimation of sugar contents.

##### Lycopene and Carotenoids

Lycopene and carotenoids in blood plasma samples were assayed according to the standardized procedure of Nagata and Yamashita (35). For estimation of lycopene and carotenoids, 1 ml of blood plasma sample was thoroughly homogenized with 10 ml of hexane and acetone solution (6:4). The assay solution was incubated for 5 min at 37°C and then filtered. The absorbance was measured at 453, 505, and 663 nm, and finally the quantities of lycopene and carotenoids were calculated with the help of the following formulae:

Lycopene = −0.0458A_663_ + 0.372A_505_

Carotenoids = 0.216A_663_ – 0.304A_505_ + 0.452A_453_

#### Hydrolytic Enzymes

##### Protease

The protease activity was measured by the casein digestion method (36). Protease enzyme releases an amino acid “tyrosine” after digestion of casein. The reaction of tyrosine Folin's reagent results in the formation of a blue color product that is quantified at 660 nm. A standard calibration curve of tyrosine was used to compute the protease activity in plasma samples. One unit of enzyme activity was defined as the amount of enzyme that causes the release of soluble acid fragments equivalent to 0.001 A 280 nm in 1 min at pH 7.8.

##### Esterase

The esterase (alpha and beta) enzyme activity was estimated by utilizing naphthyl acetate (α and β) as substrate as described by Van Asperen (37). The reaction mixture consisting of plasma (enzyme extract), phosphate buffer (0.04 M, pH 7), 30 mM naphthyl acetate (α and β), and 1% acetone was incubated in the dark at 37°C for 15 min. Then, 1 ml of staining solution composed of fast blue BB (1%) and sodium dodecyl sulfate (5%) was added in both blank control (phosphate buffer and substrate solution) and reaction mixture and again incubated for 15 min in the dark at room temperature. The absorbance of the assay was recorded at 590 nm, and the enzyme activity was calculated in μM min^−1^ ml^−1^ of a sample using a standard curve.

##### Amylase

The activity of the amylase enzyme in plasma samples was assessed by using 0.2 M tris-malate (pH 7.2) buffer as an extraction cum assay medium (38). For estimation of enzyme activity, 1 ml of substrate solution (0.15% starch) was homogenized with 1 ml of plasma and incubated at 37°C for 10 min. The OD of the reaction mixture was measured at 620 nm after adding quenching reagent and enzyme activity was represented in milligrams of starch degraded per minute per milliliter of blood plasma sample.

### Analysis of Milk

Milk samples were analyzed for chemical composition by a milk analyzer (Julie Z7, Scope Electric, Regensburg, Germany). The defatted milk samples were used for estimation of non-enzymatic (TPC, TAC, and vitamin C) and enzymatic (SOD, POD, and CAT) antioxidants by a spectrophotometer (UV-VIS U2800, Hitachi, Japan) as described above for plasma samples.

### Reproductive and Productive Performance

The reproductive and productive performance was evaluated in terms of birth weight, weight gain per week, survival rate of newly born kids, shedding time of placenta, the onset of first postpartum estrus, and milk yield in goats.

### Statistical Analysis

All the statistical analyses were carried out by using SPSS version 20. The experimental procedures were performed in triplicate, and data obtained were analyzed by one-way analysis of variance (ANOVA) with repeated measures under the shade of LSD to access the differences among different treatment means on specific days. The results were expressed in the tables as mean ± SE, and the values with *p* < 0.05 were considered statistically significant.

## Results

### Non-enzymatic Antioxidant Parameters

The response of plasma non-enzymatic antioxidant parameters to MOLP supplementation during pregnancy and early lactation period is shown in Table 4. Plasma TFs were increased significantly from day 110 of pregnancy to day 20 of lactation in the M_3.5%_ group (*p* < 0.05). The impact of supplementation was non-significant on M_2%_ group plasma TFs as compared to the control (M_0_) group (*p* > 0.05). The values of plasma TPC of the M_3.5%_ and M_2%_ groups were non-significant in the pregnancy stage, while they became significant during the early lactation period of the experiment (*p* < 0.05). The increase in plasma Vit C contents was significant (*p* < 0.05) from the 130th day of pregnancy till day 20 of lactation in both M_3.5%_ and M_2%_ supplemented groups. The MOLP supplementation significantly improved the plasma TAC by declining MDA concentration and TOS values near parturition and early lactation stage of the experiment in contrast to the control (M_0_) group (*p* < 0.05).

**Table 4:** Plasma non-enzymatic antioxidant indices of Beetal goats.

**Non-enzymatic antioxidants**	**Levels of** ***Moringa oleifera*** **leaf powder supplementation**			**SEM**	***p*** **-value**
	**M_**0**_**	**M_**2*%***_**	**M_**3.5%**_**		
Gestation day 110					
Total flavonoids (μg/ml)	239.93 ± 0.91^b^	241.06 ± 0.74^b^	253.91 ± 0.72^a^	0.43	0.030
Total phenolic contents (μM/ml)	5,821.75 ± 0.85^b^	5,832.61 ± 1.60^a^	5840 ± 2.45^a^	1.19	0.028
Vitamin C (μg/ml)	803.75 ± 1.55	805.50 ± 1.04	810.25 ± 1.44	0.84	0.221
Malondialdehyde (μM/ml)	5.90 ± 0.33	5.76 ± 0.37	5.59 ± 0.26	0.17	0.627
Total anti-oxidant capacity (μM/ml)	1.41 ± 0.02^c^	1.55 ± 0.01^b^	1.67 ± 0.01^a^	0.01	0.002
Total oxidant status (μM/ml)	1,566.25 ± 1.11	1,559.75 ± 0.63	1,555.50 ± 2.10	1.04	0.065
Gestation day 130					
Total flavonoids (μg/ml)	251.31 ± 0.59^b^	256.14 ± 0.76^b^	272.79 ± 1.07^a^	0.29	0.013
Total phenolic contents (μM/ml)	5,819 ± 0.82^b^	5,844.75 ± 1.31^a^	5,848.25 ± 0.95^a^	0.65	0.007
Vitamin C (μg/ml)	795 ± 1.08^c^	811.25 ± 1.32^b^	819.50 ± 0.96^a^	0.42	0.008
Malondialdehyde (μM/ml)	7.26 ± 0.09^b^	5.28 ± 0.23^a^	5.06 ± 0.27^a^	0.18	0.024
Total anti-oxidant capacity (μM/ml)	1.49 ± 0.01^c^	1.73 ± 0.02^b^	1.86 ± 0.02^a^	0.01	0.002
Total oxidant status (μM/ml)	1,585.25 ± 0.63^c^	1,533 ± 0.71^b^	1,502.5 ± 0.96^a^	0.39	<0.001
Gestation day 150					
Total flavonoids (μg/ml)	258.93 ± 1.13^b^	264.66 ± 0.68^b^	278.84 ± 0.58^a^	0.67	0.001
Total phenolic contents (μM/ml)	5,796.50 ± 0.86^b^	5,853 ± 0.57^a^	5,859.25 ± 2.05^a^	1.14	<0.001
Vitamin C (μg/ml)	780 ± 0.41^c^	816.75 ± 0.85^b^	827.75 ± 0.48^a^	0.39	<0.001
Malondialdehyde (μM/ml)	8.31 ± 0.12^b^	4.55 ± 0.22^a^	4.19 ± 0.18^a^	0.06	0.004
Total anti-oxidant capacity (μM/ml)	1.37 ± 0.02^c^	1.81 ± 0.01^b^	1.96 ± 0.04^a^	0.02	0.007
Total oxidant status (μM/ml)	1,605 ± 1.08^c^	1,515.75 ± 0.85^b^	1,481 ± 0.82^a^	0.86	<0.001
Lactation day 20					
Total flavonoids (μg/ml)	265.28 ± 1.23^b^	273.19 ± 2.46^b^	291.44 ± 0.63^a^	1.06	0.013
Total phenolic contents (μM/ml)	5,811.50 ± 0.64^c^	5,866 ± 0.71^b^	5,884 ± 1.08^a^	0.68	<0.001
Vitamin C (μg/ml)	783 ± 0.39^c^	825.51 ± 0.48^b^	838.69 ± 1.25^a^	0.44	<0.001
Malondialdehyde (μM/ml)	7.94 ± 0.25^c^	4.01 ± 0.04^b^	2.74 ± 0.29^a^	0.11	0.013
Total anti-oxidant capacity (μM/ml)	1.53 ± 0.01^c^	1.94 ± 0.01^b^	2.16 ± 0.04^a^	0.02	0.001
Total oxidant status (μM/ml)	1,598.50 ± 0.65^c^	1,492.50 ± 1.04^b^	1,459.75 ± 0.48^a^	0.42	<0.001

*Means with superscript letters (a, b, c) within the same row differ significantly at p ≤ 0.05*.

### Enzymatic Antioxidants

The MOLP supplementation impacts on plasma enzymatic antioxidants are presented in Table 5. The SOD activity of M_3.5%_ and M_2%_ groups increased significantly throughout the experiment in response to supplementation (*p* < 0.05) and reached its peak on day 20 of lactation in the M_3.5%_ group. The effect of supplementation levels (3.5 and 2%) was non-significant on POD activity (*p* > 0.05). However, there was a significant difference between the POD activities of supplemented (M_3.5%_ and M_2%_) and control (M_0_) groups during the entire experiment (*p* < 0.05). A non-significant increase in the plasma CAT activity was noticed till day 150 of gestation (*p* > 0.05), but soon after kidding, the CAT activity was enhanced significantly during the lactation period according to the supplementation levels in M_3.5%_ and M_2%_ groups as compared to the control (M_0_) group (*p* < 0.05).

**Table 5:** Plasma enzymatic antioxidant indices of Beetal goats.

**Enzymatic antioxidants (Units/ml)**	**Levels of** ***Moringa oleifera*** **leaf powder supplementation**			**SEM**	***p*** **-value**
	**M_**0**_**	**M_**2%**_**	**M_**3.5%**_**		
Gestation day 110					
SOD	21.29 ± 0.52^c^	25.82 ± 0.28^b^	32.08 ± 0.39^a^	0.23	0.011
POD	195.82 ± 0.61^b^	207.16 ± 0.79^a^	212.24 ± 1.30^a^	0.45	0.002
CAT	40 ± 0.82	41 ± 1.29	44.75 ± 0.48	0.52	0.062
Gestation day 130					
SOD	19.88 ± 0.63^c^	28.20 ± 0.57^b^	37.77 ± 0.73^a^	0.29	0.015
POD	209.65 ± 0.88^b^	231.79 ± 1.16^a^	238.17 ± 1.06^a^	0.34	0.001
CAT	42 ± 0.41	44.75 ± 0.85	45.25 ± 0.25	0.24	0.100
Gestation day 150					
SOD	13.76 ± 0.38^c^	28.88 ± 0.64^b^	39.54 ± 0.89^a^	0.47	0.001
POD	215.21 ± 0.74^b^	244.11 ± 0.89^a^	247.07 ± 0.98^a^	0.51	0.005
CAT	45.50 ± 0.96	47 ± 0.71	49.50 ± 0.94	0.49	0.154
Lactation day 20					
SOD	17.97 ± 0.43^c^	36.17 ± 1.05^b^	42.76 ± 0.36^a^	0.30	0.001
POD	226.91 ± 0.69^b^	261.79 ± 1.55^a^	268.11 ± 0.72^a^	0.67	0.001
CAT	50.48 ± 0.87^c^	55.50 ± 0.29^b^	63.39 ± 0.86^a^	0.38	0.023

*Means with superscript letters (a, b, c) within the same row differ significantly at p ≤ 0.05. SOD, superoxide dismutase; POD, peroxidase; CAT, catalase*.

### Biochemical Indices

The change in plasma biochemical indicators in response to MOLP supplementation is given in Table 6. The supplementation initially showed no pronounced effect on plasma TSP contents till the 130th day of pregnancy. Thereafter, the plasma TSP contents increased significantly on day 150 of pregnancy and day 20 of lactation in M_3.5%_ group in comparison to M_2%_ and M_0_ groups (*p* < 0.05). The plasma sugar level of both M_3.5%_ and M_2%_ supplemented groups increased significantly from the beginning of the experiment till day 20 of lactation (*p* < 0.05). A drastic increase in plasma sugar level was observed soon after kidding in supplemented groups in contrast to the control group. The concentration of carotenoids in plasma was significantly enhanced throughout the experiment in the M_3.5%_ group as compared to the control (M_0_) group (*p* < 0.05), whereas the lycopene contents were improved from day 130 of pregnancy to day 20 of lactation in the M_3.5%_ as well as M_2%_ group (*p* < 0.05).

**Table 6:** Plasma biochemical parameters of Beetal goats.

**Biochemicals**	**Levels of** ***Moringa oleifera*** **leaf powder supplementation**			**SEM**	***p*** **-value**
	**M_**0**_**	**M_**2%**_**	**M_**3.5%**_**		
Gestation day 110					
Total soluble proteins (mg/ml)	60.98 ± 0.73	63.87 ± 0.34	65.09 ± 1.12	0.59	0.118
Total sugars (mg/ml)	4.58 ± 0.05^c^	5.40 ± 0.04^b^	6.18 ± 0.03^a^	0.03	0.003
Carotenoids (μg/ml)	165.77 ± 0.79^b^	169.09 ± 1.07^b^	176.56 ± 0.62^a^	0.25	0.013
Lycopene (μg/ml)	98.75 ± 2.66	103 ± 1.15	108 ± 3.02	0.53	0.207
Gestation day 130					
Total soluble proteins (mg/ml)	59.56 ± 3.17	66.23 ± 0.57	69.16 ± 1.78	1.63	0.066
Total sugars (mg/ml)	4.43 ± 0.05^c^	5.80 ± 0.04^b^	6.63 ± 0.06^a^	0.02	0.004
Carotenoids (μg/ml)	170.79 ± 0.96^b^	177.45 ± 0.83^b^	185.41 ± 0.61^a^	0.49	0.001
Lycopene (μg/ml)	95 ± 1.08^c^	111 ± 0.57^b^	119.75 ± 0.85^a^	0.51	0.010
Gestation day 150					
Total soluble proteins (mg/ml)	55.96 ± 3.04^b^	61.19 ± 1.49^b^	71.02 ± 0.69^a^	1.56	0.042
Total sugars (mg/ml)	3.88 ± 0.13^c^	5.90 ± 0.04^b^	6.93 ± 0.05^a^	0.03	0.005
Carotenoids (μg/ml)	168 ± 2.19^b^	175.93 ± 0.68^b^	187.13 ± 1.30^a^	0.85	0.032
Lycopene (μg/ml)	89.75 ± 0.47^c^	116.78 ± 1.03^b^	125.69 ± 0.75^a^	0.47	0.001
Lactation day 20					
Total soluble proteins (mg/ml)	63.50 ± 1.56^b^	67.18 ± 0.35^b^	75.40 ± 0.73^a^	0.78	0.016
Total sugars (mg/ml)	4.03 ± 0.04^c^	6.30 ± 0.05^b^	7.35 ± 0.07^a^	0.04	0.002
Carotenoids (μg/ml)	172.42 ± 1.02^b^	178.74 ± 1.32^b^	193.55 ± 0.89^a^	0.73	<0.001
Lycopene (μg/ml)	93 ± 0.91^c^	118.25 ± 0.85^b^	129.5 ± 0.95^a^	0.69	<0.001

*Means with superscript letters (a, b, c) within the same row differ significantly at p ≤ 0.05*.

### Hydrolytic Enzymes

The activities of plasma hydrolytic enzymes in supplemented (M_3.5%_ and M_2%_) and control (M_0_) groups are illustrated in Table 7. The protease enzyme activity was modified more effectively from day 130 to 150 of gestation in the M_3.5%_ group in comparison to the M_2%_ group. However, the supplementation of MOLP significantly influenced plasma protease activity in the M_2%_ group during the lactation period (*p* < 0.05). However, the supplementation did not show any significant impact on esterase activity during the experiment (*p* > 0.05). The amylase activity was slightly improved in the M_3.5%_ group from day 150 of gestation in comparison to the control (M_0_) group (*p* < 0.05).

**Table 7:** Plasma hydrolytic enzymes activities of Beetal goats.

**Enzymes**	**Levels of** ***Moringa oleifera*** **leaf powder supplementation**			**SEM**	***p*** **-value**
	**M_**0**_**	**M_**2%**_**	**M_**3.5%**_**		
Gestation day 110					
Protease (U/ml)	233.25 ± 0.95	235.50 ± 0.64	239.25 ± 1.18	0.79	0.065
Esterase (μM/min/ml)	643.75 ± 0.85	641 ± 1.08	645.75 ± 0.63	0.37	0.084
Amylase (mg/min/ml)	1.31 ± 0.02	1.34 ± 0.01	1.39 ± 0.02	0.01	0.369
Gestation day 130					
Protease (U/ml)	235 ± 1.08^b^	238.50 ± 0.29^b^	243 ± 0.41^a^	0.38	0.009
Esterase (μM/min/ml)	638.25 ± 0.95	639.50 ± 0.96	641.75 ± 0.48	0.41	0.252
Amylase (mg/min/ml)	1.35 ± 0.02	1.39 ± 0.03	1.42 ± 0.01	0.02	0.110
Gestation day 150					
Protease (U/ml)	238.50 ± 0.51^b^	240.75 ± 0.75^b^	247 ± 0.82^a^	0.44	0.036
Esterase (μM/min/ml)	634.50 ± 0.87	636.50 ± 0.64	637 ± 0.91	0.25	0.486
Amylase (mg/min/ml)	1.36 ± 0.03^b^	1.43 ± 0.01^b^	1.51 ± 0.01^a^	0.01	0.036
Lactation day 20					
Protease (U/ml)	242.75 ± 0.85^c^	249.75 ± 0.63^b^	254.50 ± 0.29^a^	0.47	0.008
Esterase (μM/min/ml)	639.25 ± 0.63	642 ± 0.82	644 ± 0.56	0.33	0.059
Amylase (mg/min/ml)	1.41 ± 0.01^b^	1.44 ± 0.01^b^	1.57 ± 0.02^a^	0.04	0.016

*Means with superscript letters (a, b, c) within the same row differ significantly at p ≤ 0.05*.

### Milk Biochemical Composition

The alterations in milk biochemical composition as a result of MOLP supplementation are expressed in Table 8. The colostrum and milk protein contents were increased significantly in M_3.5%_ group. However, the supplementation in the M_2%_ group exhibited a significant impact on days 21 and 28 milk protein contents (*p* < 0.05). The milk fat percentage was not affected by supplementation in either the M_3.5%_ or M_2%_ group up to day 14 of lactation (*p* > 0.05), but on days 21 and 28, the milk fat was significantly increased in the M_3.5%_ group as compared to the control (M_0_) group (*p* < 0.05). There was no influence of supplementation on the lactose contents of colostrum and mature milk samples of M_3.5%_ and M_2%_ groups (*p* > 0.05). Total carotenoids were significantly high in the milk samples of the M_3.5%_ group as compared to M_2%_ and M_0_ groups from day 0 to day 28 of lactation (*p* < 0.05).

**Table 8:** Effect of *Moringa oleifera leaf powder* supplementation on milk composition.

**Biochemical**	**Levels of** ***Moringa oleifera*** **leaf powder supplementation**			**SEM**	***p*** **-value**
	**M_**0**_**	**M_**2**%****_**	**M_**3.5%**_**		
Day 0 (Colostrum)					
Protein (%)	8.93 ± 0.37^b^	10.18 ± 0.28^b^	13.53 ± 0.53^a^	0.24	0.043
Fat (%)	8.95 ± 0.40	9.18 ± 0.48	9.53 ± 0.34	0.37	0.092
Lactose (%)	2.93 ± 0.29	3.09 ± 0.31	3.27 ± 0.13	0.21	0.663
Carotenoids (μg/ml)	15.22 ± 0.83^b^	16.71 ± 0.40^b^	18.53 ± 0.39^a^	0.49	0.031
Day 7					
Protein (%)	6.84 ± 0.09^b^	7.11 ± 0.31^b^	8.51 ± 0.25^a^	0.16	0.015
Fat (%)	7.38 ± 0.86	7.94 ± 0.62	8.72 ± 0.30	0.61	0.233
Lactose (%)	3.43 ± 0.07	3.62 ± 0.21	3.71 ± 0.16	0.05	0.514
Carotenoids (μg/ml)	6.91 ± 0.19^b^	7.36 ± 0.07^b^	9.82 ± 0.36^a^	0.15	0.041
Day 14					
Protein (%)	5.08 ± 0.36^b^	5.40 ± 0.25^b^	7.94 ± 0.46^a^	0.28	0.045
Fat (%)	6.56 ± 0.35	6.87 ± 0.39	7.19 ± 0.52	0.28	0.645
Lactose (%)	3.97 ± 0.14	4.01 ± 0.23	4.14 ± 0.20	0.09	0.273
Carotenoids (μg/ml)	4.68 ± 0.41^b^	4.94 ± 0.16^b^	6.37 ± 0.08^a^	0.2	0.022
Day 21					
Protein (%)	4.03 ± 0.13^c^	5.15 ± 0.08^b^	6.56 ± 0.20^a^	0.08	0.012
Fat (%)	4.94 ± 0.34^b^	5.32 ± 0.11^b^	6.29 ± 0.16^a^	0.17	0.024
Lactose (%)	4.37 ± 0.51	4.51 ± 0.58	4.89 ± 0.78	0.1	0.748
Carotenoids (μg/ml)	2.73 ± 0.21^b^	3.02 ± 0.13^b^	4.36 ± 0.11^a^	0.09	0.007
Day 28					
Protein (%)	3.43 ± 0.19^c^	4.27 ± 0.11^b^	5.82 ± 0.40^a^	0.22	0.027
Fat (%)	4.87 ± 0.13^b^	3.76 ± 0.15^b^	3.41 ± 0.02^a^	0.09	0.003
Lactose (%)	4.95 ± 0.80	5.08 ± 0.19	5.35 ± 0.49	0.15	0.917
Carotenoids (μg/ml)	1.38 ± 0.36^b^	1.61 ± 0.25^b^	3.04 ± 0.08^a^	0.17	0.025

*Means with superscript letters (a, b, c) within the same row differ significantly at p ≤ 0.05*.

### Milk Enzymatic and Non-enzymatic Antioxidants

The enzymatic and non-enzymatic antioxidant parameters of the colostrum and milk samples are presented in Table 9. The TPCs in the colostrum and milk samples of the M_3.5%_ group were significantly increased in response to supplementation. There was no effect of supplementation on the TPCs of colostrum from the M_2%_ group, while these TPCs were improved significantly in the milk samples of the M_2%_ group from day 14 to 28 of lactation (*p* < 0.05). The TAC of the milk from M_3.5%_ and M_2%_ groups was significantly increased from the beginning to day 28 of lactation in comparison to the control (M_0_) group (*p* < 0.05). The significant impact of MOLP on colostrum and milk Vit C level was noticed only in the M_3.5%_ group (*p* < 0.05). Both the supplementation levels (3.5 and 2%) showed a significant impact on the SOD activity of colostrum and milk samples as compared to the non-supplemented (M_0_) group (*p* < 0.05). Initially, the POD activity was increased significantly irrespective of the level of supplementation. However, from day 21 to 28 of lactation, a significant difference was observed in the improvement of POD activity depending on levels of supplementation. The increase in the activity of CAT enzyme was non-significant up to day 14 of lactation in both M_3.5%_ and M_2%_ groups (*p* > 0.05). However, on days 21 and 28, the supplementation resulted in a significant enhancement in the CAT activity of milk samples from the M_3.5%_ group in comparison to the control (M_0_) group (*p* < 0.05).

**Table 9:** Effect of *Moringa oleifera leaf powder* supplementation on milk antioxidant parameters.

**Non-enzymatic antioxidants**	**Levels of** ***Moringa oleifera*** **leaf powder supplementation**			**SEM**	***p*** **-value**
	**M_**0**_**	**M_**2%**_**	**M_**3.5%**_**		
Day 0 (Colostrum)					
Total phenolic contents (μM/ml)	2,826.75 ± 3.49^b^	2,838.50 ± 0.64^b^	2,875.50 ± 1.55^a^	1.06	0.002
Total anti-oxidant capacity (μM/ml)	0.92 ± 0.02^c^	1.42 ± 0.08^b^	1.78 ± 0.13^a^	0.07	0.005
Vitamin C (μg/ml)	457.75 ± 1.65^b^	464.75 ± 1.79^b^	483 ± 0.82^a^	0.97	0.005
SOD (Units/ml)	69.86 ± 0.46^c^	88.65 ± 0.69^b^	96.53 ± 0.32^a^	0.18	0.001
POD (Units/ml)	291.25 ± 0.63^b^	309.22 ± 0.85^a^	314.02 ± 1.04^a^	0.37	0.003
CAT (Units/ml)	71.75 ± 0.85	76.75 ± 1.11	84 ± 1.78	1.01	0.060
Day 7					
Total phenolic contents (μM/ml)	2,608.85 ± 2.38^b^	2,622 ± 0.71^b^	2,669.25 ± 1.88^a^	0.21	0.005
Total anti-oxidant capacity (μM/ml)	0.81 ± 0.05^c^	1.39 ± 0.12^b^	1.73 ± 0.11^a^	0.09	0.016
Vitamin C (μg/ml)	421.75 ± 1.97^b^	427.25 ± 1.32^b^	436.75 ± 0.75^a^	0.78	0.035
SOD (Units/ml)	75.75 ± 0.63^c^	81.52 ± 0.26^b^	94.4 ± 0.39^a^	0.16	0.001
POD (Units/ml)	263.45 ± 0.87^b^	277.56 ± 1.32^a^	285 ± 1.65^a^	0.28	0.015
CAT (Units/ml)	66.75 ± 1.37	69.75 ± 1.18	74 ± 0.41	0.35	0.103
Day 14					
Total phenolic contents (μM/ml)	2,471.50 ± 1.32^c^	2,493.25 ± 1.03^b^	2,547 ± 2.55^a^	1.03	<0.001
Total anti-oxidant capacity (μM/ml)	0.74 ± 0.10^c^	1.29 ± 0.13^b^	1.64 ± 0.12^a^	0.12	0.017
Vitamin C (μg/ml)	412.70 ± 1.31^b^	416.40 ± 1.28^b^	429.60 ± 0.93^a^	0.71	0.016
SOD (Units/ml)	61.52 ± 0.31^c^	74.40 ± 0.41^b^	83.45 ± 0.55^a^	0.33	<0.001
POD (Units/ml)	251.67 ± 0.79^b^	270.92 ± 1.37^a^	276.05 ± 0.96^a^	0.19	0.008
CAT (Units/ml)	50.61 ± 1.51	56.75 ± 1.43	62 ± 1.92	0.69	0.072
Day 21					
Total phenolic contents (μM/ml)	2,289 ± 1.47^c^	2,326 ± 0.91^b^	2,391.50 ± 0.65^a^	0.32	0.001
Total anti-oxidant capacity (μM/ml)	0.65 ± 0.04^c^	1.24 ± 0.11^b^	1.53 ± 0.09^a^	0.08	0.013
Vitamin C (μg/ml)	385.75 ± 2.46^b^	391.50 ± 1.50^b^	408.25 ± 0.75^a^	0.57	0.021
SOD (Units/ml)	58.65 ± 0.69^c^	63.45 ± 0.91^b^	75.04 ± 0.48^a^	0.51	0.003
POD (Units/ml)	227.02 ± 1.08^c^	243.01 ± 0.56^b^	259.66 ± 0.73^a^	0.25	0.008
CAT (Units/ml)	37.95 ± 1.31^b^	41.98 ± 0.32^b^	53.30 ± 0.61^a^	0.21	0.007
Day 28					
Total phenolic contents (μM/ml)	2,133 ± 0.82^c^	2,173.25 ± 1.70^b^	2,205.75 ± 1.25^a^	0.59	<0.001
Total anti-oxidant capacity (μM/ml)	0.61 ± 0.13^c^	1.11 ± 0.04^b^	1.37 ± 0.07^a^	0.07	0.007
Vitamin C (μg/ml)	374.45 ± 1.04^b^	378.70 ± 0.85^b^	395.25 ± 0.69^a^	0.52	0.001
SOD (Units/ml)	51.70 ± 0.77^c^	59.65 ± 1.05^b^	66 ± 0.41^a^	0.57	0.003
POD (Units/ml)	202.66 ± 0.68	216.02 ± 1.12	231.01 ± 1.31	0.55	0.006
CAT (Units/ml)	29.75 ± 0.43^b^	31.36 ± 0.59^b^	46 ± 0.42^a^	0.26	0.002

*Means with superscript letters (a, b, c) within the same row differ significantly at p ≤ 0.05. SOD, superoxide dismutase; POD, peroxidase; CAT, catalase*.

### Productive and Reproductive Performance

**Figure 1:**
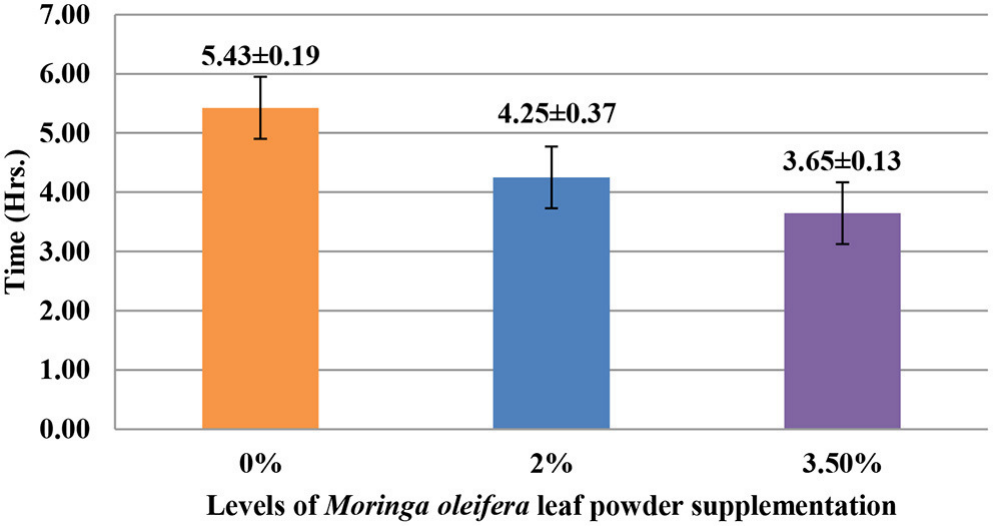
Effect of *Moringa oleifera* leaf powder supplementation on shedding time (hours) of placenta in Beetal goats.

**Figure 2:**
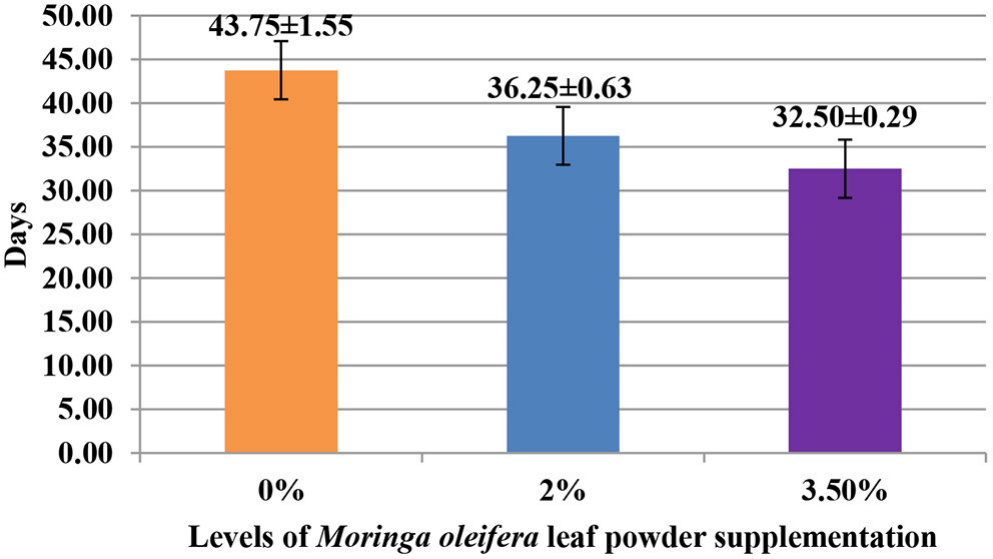
Effect of *Moringa oleifera* leaf powder supplementation on onset time (days) of first postpartum estrus after kidding in Beetal goats.

**Figure 3:**
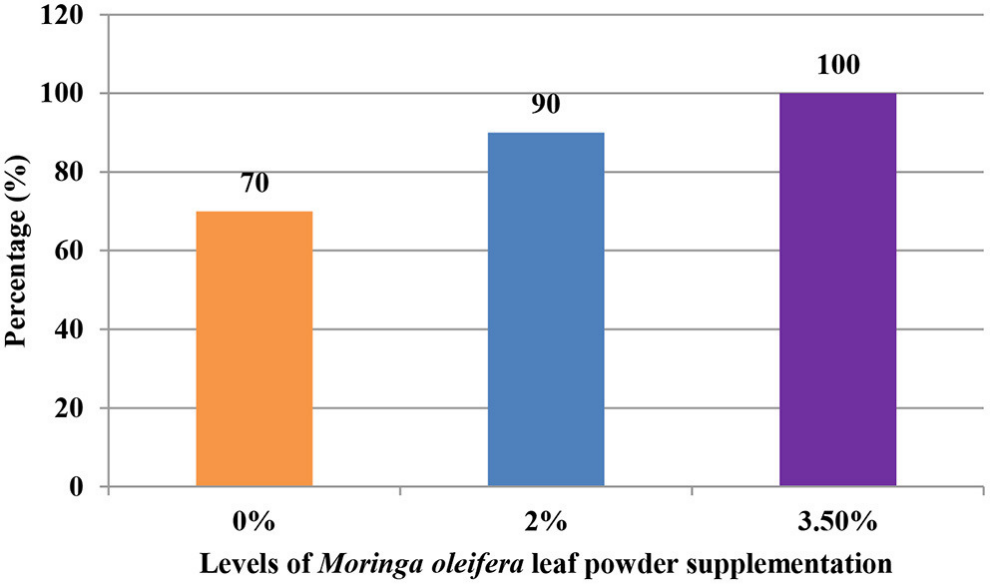
Survival rate (%) of kids in response to milk feeding of goats supplemented with *Moringa oleifera* leaf powder.

**Figure 4:**
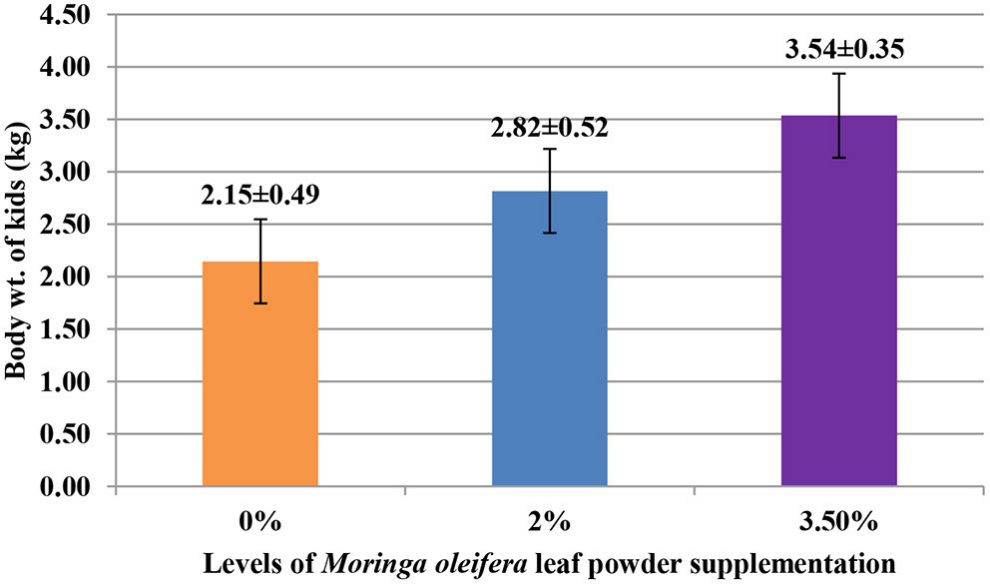
Effect of *Moringa oleifera* leaf powder supplementation on birth weight (kg) of kids in Beetal goats.

The supplementation of MOLP increased the milk production in M_3.5%_ and M_2%_ groups as compared to the control (M_0_) group from day 7 to 28 of lactation (*p* < 0.05) as is presented in Table 10. Similarly, the weight gain of kids in supplemented groups was significantly high (Table 11). The results of reproductive parameters (Figures 1, 2) showed that the shedding time of the placenta and the time of onset of first postpartum estrus was less in the goats of M_3.5%_ and M_2%_ groups as compared to the goats of the (M_0_) control group. The survival rate and initial birth weight of the kids of M_3.5%_- and M_2%_-supplemented goats were higher than M_0_ group goats (Figures 3, 4).

**Table 10:** Effect of *Moringa oleifera leaf powder* supplementation on milk production (liters) in Beetal goats.

**Days**	**Levels of** ***Moringa oleifera*** **leaf powder supplementation**			**SEM**	***p*** **-value**
	**M_**0**_**	**M_**2%**_**	**M_**3.5%**_**		
07	1.01 ± 0.03^c^	1.27 ± 0.05^b^	1.41 ± 0.04^a^	0.04	0.007
14	1.32 ± 0.14^c^	1.48 ± 0.11^b^	1.65 ± 0.09^a^	0.11	0.033
21	1.54 ± 0.07^c^	1.73 ± 0.06^b^	2.01 ± 0.07^a^	0.06	0.031
28	1.87 ± 0.16^c^	2.11 ± 0.13^b^	2.72 ± 0.15^a^	0.14	0.04

*Means with superscript letters (a, b, c) within the same row differ significantly at p ≤ 0.05*.

**Table 11:** Effect of feeding *Moringa oleifera* leaf powder supplemented goat milk on body weight (kg) of their kids.

**Days**	**Levels of** ***Moringa oleifera leaf powder*** **supplementation**			**SEM**	***p*** **-value**
	**M_**0**_**	**M_**2%**_**	**M_**3.5%**_**		
0	2.14 ± 0.49^c^	2.81 ± 0.52^b^	3.53 ± 0.36^a^	0.45	0.037
7	2.91 ± 0.17^c^	3.77 ± 0.32^b^	4.71 ± 0.28^a^	0.25	0.016
14	3.64 ± 0.22^c^	4.51 ± 0.29^b^	5.88 ± 0.12^a^	0.2	0.001
21	4.49 ± 0.42^c^	5.72 ± 0.18^b^	6.96 ± 0.14^a^	0.24	0.021
28	5.03 ± 0.16^c^	6.61 ± 0.24^b^	8.36 ± 0.35^a^	0.16	0.044

*Means with superscript letters (a, b, c) within the same row differ significantly at p ≤ 0.05*.

## Discussion

The animals are more prone to oxidative stress during the transition period because an increase in energy requirements to support developing fetuses and subsequent lactation coincide with depressed feed intake. The nutritional quality of feedstuffs is very important to regulate the pregnancy and lactation performance of goats. The MO leaves are well-known for their nutritional and therapeutic properties that have been attributed to their various phytochemical constituents (39, 40).

The pregnancy and lactation stress results in the excessive production of hydroxyl (OH) and nitric oxide (NO) radicals. The animal body is well-equipped with different protective mechanisms to neutralize the harmful effects of OH and NO radicals. The antioxidant protective system is naturally suppressed near parturition due to certain physiological changes in the body (41). The MOLP supplementation to pregnant goats resulted in an increase in their plasma TFs and TPCs that control the immense production of OH and NO radicals *via* Haber-Weiss and Fenton reactions to minimize the detrimental effects of oxidative stress during pregnancy and lactation (42). The regulation of antioxidant defense system of the body under different stress conditions through MO leaf extract supplementation was also reported in rats (43).

Vitamin C is generally regarded as the first line of defense to provide protection from the damaging effects of oxidative stress in pregnancy especially near parturition (44). A constant decline in plasma vit C level near parturition aggravates the situation that makes the animals more vulnerable to oxidative damage (45). A significant improvement in plasma vit C level during this study in supplemented goats showed that MOLP as a rich source of vit C has the ability to protect the pregnant goats from the deleterious effects of oxidative stress by suppressing the action of free radicals. The increase in plasma vit C concentration in response to MOLP supplementation is in accordance with the findings of a study on Aardi goats (14).

Lipids act as substrate for reactive nitrogen and oxygen species (RNS and ROS) to start the process of lipid peroxidation (LPO) (46, 47). The balance between the production and elimination of ROS and RNS from the body is sustained by antioxidant defense system in healthy animals. Any untoward disturbance in this balance may enhance the plasma TOS and MDA concentration. The high values of TOS and MDA represent the state of oxidative stress (48). It is evident from the results of this study that the supplementation of MOLP increased the plasma TAC in treated goats by suppressing the process of LPO. This consequently reduces the values of plasma TOS and MDA. The enzymatic (SOD, POD, and CAT) antioxidants also have a key role in limiting LPO, as both enzymatic and non-enzymatic components of antioxidant defense system work in collaboration to maintain the conditions suitable for mother and developing fetus by minimizing the parturition and early lactation stress (49).

The physiological changes in maternal body during the transition period especially near parturition result in excessive production of ROS (50). These ROS are converted into H_2_O_2_ by SOD enzyme, while POD and CAT enzymes further degrade the H_2_O_2_ into water and molecular oxygen (51, 52). The successful completion of parturition stage and start of healthy lactation depend on the activities of SOD, POD, and CAT enzymes (53). High antioxidant (SOD, POD, and CAT) enzyme activities indicate that the supplementation of MOLP in late pregnancy improved the plasma enzymatic antioxidants that was also reported in some other studies conducted with different supplementation levels of MO in rabbits, poultry, and dairy cows (54–56).

Blood biochemical parameters are the established indicators to provide information about the health status of pregnant animals for their successful transfer from gestation to lactation stage (57). Late pregnancy is characterized by severe metabolic changes and a rapid decrease in plasma protein level was also noticed in different other animal species during this period (58–60). The plasma protein level drops promptly in the last trimester due to high amino acid requirements for developing fetus and preparation of the mammary system for subsequent lactation stage (61). However, after parturition, the plasma protein level starts to increase due to immense production of immunoglobulins (62). It is evident from the findings of this study that the plasma protein level was high in supplemented groups as compared to the control group of the experiment during the peripartum period. The supplementation of MOLP increased the plasma protein contents to satisfy the high protein requirements of pregnancy and lactation. These results support the findings of other studies in Jersey cattle and sows (21, 63). The presence of high amount of protein in MOLP enhances the synthesis of selenocysteine-based selenoproteins. These proteins have been reported to play a role in the modification of antioxidant defense system and improvement of reproductive functions (64).

The decrease in plasma sugar level near parturition is typical for goats and ewes. A reduction in feed intake occurs during late pregnancy due to the squeezing of the rumen by rapid fetal growth (65). The mobilization of the body fats starts if the energy requirements of the animals are not fulfilled with the advancement in pregnancy through provision of appropriate feed supplement (66). The negative energy balance in this stage may lead to the development of ketosis and some other metabolic diseases. The feeding of MOLP increased the plasma sugar level of supplemented groups to provide sufficient amount of energy for parturition and early lactation. The revival of gluconeogenesis process after parturition in response to certain endocrine changes rapidly increase the plasma sugar level (67, 68). Furthermore, the supplementation also improved the plasma lycopene and carotenoids status to regulate the synthesis of inflammatory cytokines that reduces the chances of complications at the time of parturition (69, 70).

A proteolytic enzyme system in the body helps in the removal of worthless and damaged biomolecules to maintain hemostasis during pregnancy and lactation (71). The significant increase in the activity of protease enzyme in MOLP-supplemented groups depicted its defensive effects at the cellular level. The proteolytic enzyme system is of great importance because it also has the ability to act as a secondary antioxidant defense system when the primary antioxidant system is unable to protect the body from oxidative stress (72). The supplementation of MOLP also resulted in an improvement in the amylase activity. This enzyme enhances the conversion of carbohydrates into glucose to produce energy according to the requirements of body during pregnancy and lactation (66, 73).

The major outcome of the study was that supplementing the goats' ration with MOLP markedly influenced their colostrum and milk composition. The preparations for colostrum synthesis start in the last month of pregnancy (74). Colostrum provides energy and maternal immunity along with different other growth factors to maintain health status and development of newly born kids. The findings of this study showed that supplementation of MOLP increased the protein contents of colostrum and milk in supplemented groups. The concentration of protein is generally high in colostrum than normal milk due to the presence of Igs in huge amount (75). The consumption of Igs protects the newly born kids from different diseases and thereby enhances their survival rate. The supplementation also improved the fat percentage of mature milk, which is in accordance with the findings of Kholif et al. (76). MOLP stimulates the production of acetate that acts as a major precursor for the biosynthesis of fat (77). Similarly, a significant increase in carotenoids of colostrum and milk was also noticed with 3.5% supplementation. The concentration of carotenoids in milk mostly depends on the type of feed and MOLP being a rich source of carotenoids has positive effects on the carotenoid contents of colostrum and milk in both supplemented groups. The carotenoids have been reported to play an important role in the improvement of milk quality by preventing the process of auto-oxidation (78).

The presence of antioxidants in appropriate amount prolongs the shelf life of milk and reduces its chances of microbial spoilage. The antioxidants in milk also protect the suckling kids from various health hazards by strengthening their immune system (79). The supplementation of diet with MOLP increased the colostrum and milk TAC as was also reported previously in cows by Kekana et al. (63). This could be due to the synergistic effects of flavonoids, phenolics, Se, and vit C present in MOLP. Generally, milk is not considered a good source of vit C. However, the results of this study showed the presence of an appreciable amount of vit C in the milk of goats supplemented with MOLP. Thus, feeding the goats with MOLP-supplemented diet positively influenced the vit C contents in their milk. The presence of functional antioxidants in MOLP also improved the enzymatic (SOD, POD, and CAT) antioxidant status of the milk to fulfill the demands of both milk producers and consumers for healthier dairy products. The favorable impacts of MO supplementation on milk composition were also reported in some other studies performed on dairy animals (80, 81).

The beneficial effects of MOLP supplementation on reproductive performance parameters observed in this study were due to its high nutritional profile. The birth weight of kids in supplemented groups was high as compared to the control group. The supplementation of MOLP in advance stage of pregnancy increases the provision of protein to developing fetus for its further growth and also improves the protein contents of colostrum and milk (82). The high level of protein contents in the milk of supplemented goats promoted the weight gain in their kids. The presence of therapeutic compounds in MOLP increased the survival rate of the kids of supplemented goats by protecting them from various diseases (83). Similar findings were reported by Qwele et al. (84), who disclosed that feeding of MO-supplemented diet is beneficial for the animals to protect them from oxidative stress-induced diseases. High milk production in early lactation stage often results in negative energy balance (NEB), if the nutrient supply is inadequate to lactating animals. The NEB is the major cause of delay in shedding of placental membranes and resumption of postpartum estrus after parturition (85). MOLP supplementation improved the energy status of the body to ensure the revival of ovarian activity and reduces postpartum anestrus interval. The shortening of postpartum anestrus duration by dietary modifications has also been reported previously in cows (86). The high proportion of protein and Se contents in MO strengthened the uterine muscle contractions for timely shedding of placenta and thus protects the reproductive tract from different infections.

## Conclusion

The results of the current study revealed that supplementing 3.5% MOLP improved maternal health and milk quality in terms of antioxidant status and biochemical composition. Furthermore, this supplementation level also increased the milk yield, kids' growth rate, and reproductive performance of goats. These findings propose that MOLP has the potential to improve the productive/reproductive performance of goats. However, further studies are required with different feeding levels of MOLP to explore the molecular aspects of improving productive and reproductive performance in large herds of animals.

## Author's Note

This experiment/research paper is a part of Ali Afzal PhD study.

## Data Availability Statement

The raw data supporting the conclusions of this article will be made available by the authors, without undue reservation.

## Ethics Statement

The procedures used for study and ethical clearance was approved and granted by Animal Use and Care Research Committee at Nuclear Institute for Agriculture and Biology (NIAB), Faisalabad, Pakistan.

## Author Contributions

AA: conduct experiment, data aggregation, statistical analysis, and wrote manuscript. AH: lab analysis. TH: design experiment, monitoring experiment, and revised manuscript. MS and MM: methodology. GY: manuscript editing and revision. All authors contributed to the article and approved the submitted version.

## Conflict of Interest

The authors declare that the research was conducted in the absence of any commercial or financial relationships that could be construed as a potential conflict of interest.

## Publisher's Note

All claims expressed in this article are solely those of the authors and do not necessarily represent those of their affiliated organizations, or those of the publisher, the editors and the reviewers. Any product that may be evaluated in this article, or claim that may be made by its manufacturer, is not guaranteed or endorsed by the publisher.
